# Evolution and Conservation of Plant NLR Functions

**DOI:** 10.3389/fimmu.2013.00297

**Published:** 2013-09-25

**Authors:** Florence Jacob, Saskia Vernaldi, Takaki Maekawa

**Affiliations:** ^1^Department of Plant-Microbe Interactions, Max Planck Institute for Plant Breeding Research, Cologne, Germany; ^2^Unité de Recherche en Génomique Végétale, Institut National de la Recherche Agronomique, Centre National de la Recherche Scientifique, Université Evry Val d’Essone, Evry, France

**Keywords:** NLR, NB-LRR, resistance protein, innate immunity, effector-triggered immunity

## Abstract

In plants and animals, nucleotide-binding domain and leucine-rich repeats (NLR)-containing proteins play pivotal roles in innate immunity. Despite their similar biological functions and protein architecture, comparative genome-wide analyses of *NLRs* and genes encoding NLR-like proteins suggest that plant and animal NLRs have independently arisen in evolution. Furthermore, the demonstration of interfamily transfer of plant NLR functions from their original species to phylogenetically distant species implies evolutionary conservation of the underlying immune principle across plant taxonomy. In this review we discuss plant NLR evolution and summarize recent insights into plant NLR-signaling mechanisms, which might constitute evolutionarily conserved NLR-mediated immune mechanisms.

## Introduction

Plants rely entirely on innate immunity to fight pathogens (1), as they do not have an adaptive immune system, including specialized immune cells, like higher animals. To achieve a specialized and targeted immune response, plants possess several lines of defense against pathogens. Plasma membrane localized pattern-recognition receptors recognize conserved pathogen molecules, such as flagellin and chitin and provide broad-spectrum pathogen resistance (2). However, host-adapted pathogens suppress this immune response by delivering effector molecules inside host cells (3, 4). As a counter mechanism, plants deploy the nucleotide-binding domain and leucine-rich repeats (NLR) family of intracellular receptors to detect the presence of effectors, triggering potent innate immune responses (5, 6). The former class of immunity is called “pattern-triggered immunity” (PTI), whereas the latter is called “effector-triggered immunity” (ETI), which is often associated with genetically programed host cell death (1).

The mechanism of effector recognition by plant NLRs has been well established. Plant NLRs utilize two major modes of effector recognition: a direct and an indirect recognition mode (5–8). In both cases, plant NLRs are kept in an inactive form by either intra- or inter-molecular interactions in the absence of cognate effectors (9). The difference lies within the mode of effector recognition: in case of the direct recognition, an effector is detected by direct physical interaction with its cognate NLR, whereas during the indirect recognition, a NLR senses modifications of host proteins caused by the cognate effector action. Experimental evidence supports that the indirect recognition enables a single NLR to recognize multiple effectors irrespective of effector structures when effectors target the same host protein (5, 6). However, detection of multiple effectors by a single NLR is not exclusive to the indirect recognition mode. Recently it was demonstrated that a single NLR can detect at least two sequence-unrelated effectors by direct binding (10).

Knowledge on signal initiation and transduction mediated by plant NLRs is rather sparse compared to the effector detection mechanism. However, through recent progress in plant NLR biology, the mechanisms of signal initiation and signaling relay are gradually being revealed. Furthermore, the demonstration of interfamily transfer of NLR functions across plant lineages implies evolutionary conservation of the underlying immune mechanisms. On the following pages, we will discuss plant NLR evolution and summarize recent insights into plant NLR-signaling mechanisms, which might hint at yet unidentified, evolutionarily conserved NLR-mediated immune signaling mechanisms. Furthermore, comparative genome-wide analyses of genes encoding NLRs and NLR-like proteins among various plant lineages give insights into the presumed history of plant NLR evolution and consequently important clues to elucidate NLR functions in innate immunity and possibly functions beyond innate immunity.

## Survey of *NLR* Genes in Land Plants: Toward a Model of Plant NLR Evolutionary History

### Expanded *NLR* repertoires across plant lineages

Similar to animal NLRs, plant NLRs are modular proteins that generally consist of three building blocks: a N-terminal domain, the central NB-ARC domain (named after Nucleotide-Binding adaptor shared with APAF-1, plant resistance proteins, and CED-4), and a C-terminal LRR (leucine-rich repeats) domain (11). The central domain of animal NLRs is also known as the NACHT domain (named after NAIP, CIITA, HET-E, and TP1) (12) which is structurally similar to the plant NB-ARC domain but distinctive of animal NLRs (13, 14). The utilization of either a TOLL/interleukin 1 receptor (TIR) domain or a coiled-coil (CC) domain at the N-terminus is a plant-NLR-specific feature and defines two major types of plant NLRs termed the TIR-type NLRs (TNLs) and the CC-type NLRs (CNLs), respectively. However, it is often challenging to specify structures of N-terminal domains for a significant proportion of plant NLRs due to their structural diversity and lack of significant homology to validated protein structures. Thus, NLRs containing an N-terminus other than the TIR domain are sometimes designated as non-TIR-type NLRs (nTNLs) as a distinction to TNLs.

**Figure 1:**
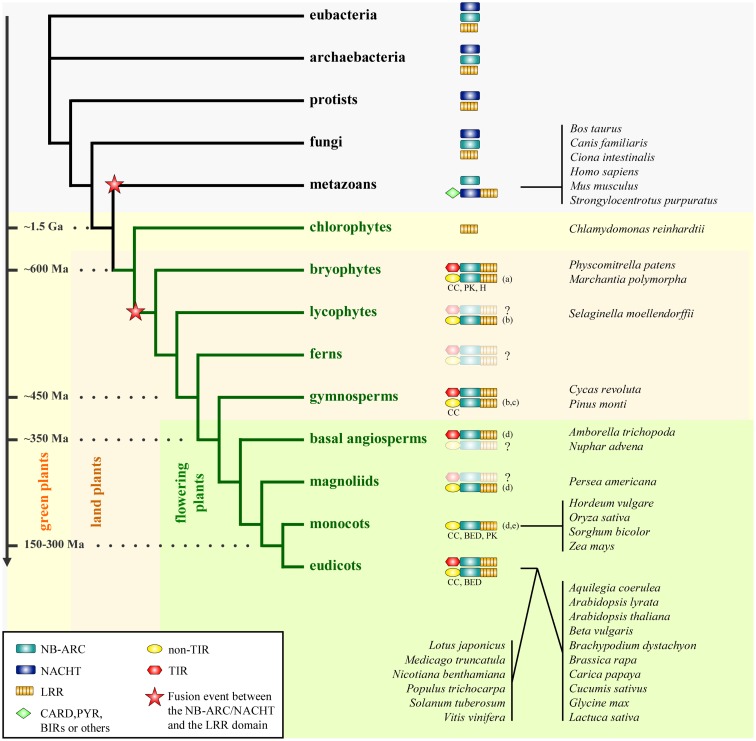
**Phylogenetic distribution of the NLR family**. The distribution of the individual domains constitutive of NLRs (NB-ARC, NACHT, and LRR) and the different groups of NLRs are mapped on a simplified phylogenetic tree. The fusion events between either the NB-ARC or the NACHT domain and the LRR domain presumably occurred as indicated on the phylogenetic tree. The structural properties of the N-termini of plant NLRs in the non-TIR group are indicated if the information is available (CC, coiled-coil; BED, BED-DNA-binding zinc finger; H, α/β-hydrolase; PK, protein kinase; for more detail, see Atypical Domains Found in the NLR Structure). This figure is adapted from Yue et al. (25), combined with data as indicated below. The divergence dates are adapted from Ref. (26) and (27). Species representative of some taxa are indicated on the right. Ma, million years; Ga, billion years. The question mark (?) indicates that the presence of NLRs is not clearly resolved in given taxa due to lack of data. (a) Xue et al. (28), (b) Kim et al. (29), (c) Heller et al. (30), (d) Tarr and Alexander (31), (e) Faris et al. (32).

The NLR family has massively expanded in several plant species. The massive expansions render the NLR family one of the largest and most variable plant protein families (15, 16). This contrasts with the vertebrate *NLR* repertoires, typically comprising ca. 20 members (17–20). Detailed genome-wide surveys, database mining, and degenerate PCR approaches for the species whose genome sequences are currently not available contribute to refine an overview of the NLR repertoires in various plant species (Table 1). Most of the plant genomes surveyed so far have a large *NLR* repertoire with up to 459 genes in wine grape (Table 1). Interestingly, the bryophyte *Physcomitrella patens* and the lycophyte *Selaginella moellendorffii* which represent the ancestral land plant lineages seem to have a relatively small *NLR* repertoire of ∼25 and ∼2 *NLRs* respectively, suggesting that the gene expansion has occurred mainly in flowering plants (Table 1; Figure 1). It was recently shown that numerous microRNAs target nucleotide sequences encoding conserved motifs of NLRs (e.g., P-loop) in many flowering plants (21). Thus it is hypothesized that such a bulk control of *NLR* transcripts may allow a plant species to maintain large *NLR* repertoires without depletion of functional *NLR* loci (22, 23), since microRNA-mediated transcriptional suppression of *NLR* transcripts could compensate for the fitness costs related to maintenance of *NLRs* (21, 24).

**Table 1:** **Plant *NLR* gene repertoires identified by genome-wide analyses**.

Species	Common name	Genome size (Mbp)	*NLRs*	*TNLs*	*CNLs*	*XNLs*	Reference
*Arabidopsis thaliana*	Thale cress	125	151	94	55	0	Meyers et al. (18)
*Arabidopsis lyrata*	Lyre-leaved rock-cress	230	138	103	21	NA	Guo et al. (33)
*Brachypodium distachyon*	Brachypodium	355	212	0	145	60	Li et al. (34)
*Brassica rapa*	Mustard	100–145^a^ (529)	80	52	28	NA	Mun et al. (35)
*Carica papaya*	Papaya	372	34	6	4	1	Porter et al. (36)
*Chlamydomonas reinhardtii*	Chlamydomonas	120	0	0	0	0	Yue et al. (25)
*Cucumis sativus*	Cucumber	367	53	11	17	2	Wan et al. (37)
*Glycine max*	Soybean	1115	319	116	20	NA	Kang et al. (38)
*Medicago truncatula*	Barrel medic	186^a^ (500)	270	118	152	0	Ameline-Torregrosa et al. (39)
*Oryza sativa*	Rice	466	458	0	274	182	Li et al. (34)
*Physcomitrella patens*	Moss	511	25	8	9	8	Xue et al. (28)
*Populus trichocarpa*	Poplar	550	317	91	119	34	Kohler et al. (40)
*Sorghum bicolor*	Sorghum	760	184	0	130	52	Li et al. (34)
*Solanum tuberosum*	Potato	840	371	55	316	NA	Jupe et al. (41)
*Selaginella moellendorffii*	Spike moss	100	2	0	NA	NA	Yue et al. (25)
*Vitis vinifera*	Wine grape	487	459	97	215	147	Yang et al. (42)
*Zea mays*	Maize	2400	95	0	71	23	Li et al. (34)

The table represents *NLR* and *NLR*-like gene numbers corresponding to NB-ARC-LRR-encoding genes. The numbers for *TNLs*, *CNLs*, and *XNLs* correspond to genes encoding either full-length TNLs, CNLs, XNLs, or the NB-ARC-LRR-containing proteins if these can be clearly assigned to one of the NLR types based on their motif composition at the NB-ARC domain. X refers to any N-terminal domain other than TIR or CC. ^a^Analyses based on partial genome sequence; the respective complete genome sizes are indicated in brackets.

The number of *NLR* genes in flowering plants is largely variable without any clear correlation to the phylogeny, suggesting species-specific mechanisms in *NLR* genes expansion and/or contraction (Table 1). This variability can be exemplified by three species in the brassicaceae family: *Arabidopsis thaliana*, *Arabidopsis lyrata*, and *Brassica rapa*, which have 151, 138, and 80 full-length *NLRs*, respectively (Table 1). Expansion of *NLR* genes has also occurred in several metazoans such as sea urchin (*Strongylocentrotus purpuratus*) and sea squirt (*Ciona intestinalis*), which possess 206 and 203 *NLRs*, respectively (20, 43, 44). In contrast, the genomes of fruit fly (*Drosophila melanogaster*) and nematode (*Caenorhabditis elegans*) apparently lack *NLRs*, suggesting that *NLRs* have been lost in these invertebrate species (17).

### Origin of NLR building blocks

Comparison of *NLR* repertoires from higher plants to ancestral taxa common for plants and animals could hint at the time and mechanism which led to the assembly of NLR building blocks into a single multi-domain receptor. Yue et al. (25) conducted a full genome-wide comparison of *NLR* repertoires among 38 model organisms encompassing all the major taxa (6 eubacteria, 6 archaebacteria, 6 protists, 6 fungi, 7 plants, and 7 metazoans). This dataset was further enriched with the genomic and transcriptomic data available for 5,126 species of nine major early plant lineages (chlorokybales, klebsormidiales, zygnematales, coleochaetales, charales, liverworts, bryophytes, hornworts, and lycophytes). The results of this large-scale data mining imply that the core building blocks of NLRs, such as NB-ARC, NACHT, TIR, and LRR, already existed before eukaryotes and prokaryotes diverged, since these constitutive domains are also found in the genomes of eubacteria and archaebacteria surveyed (Figure 1).

### Independent fusion events in the early history of animal and plant NLRs

The aforementioned study implies that the fusion events between an ancestral NACHT domain and an LRR domain, and between an ancestral NB-ARC domain and an LRR domain occurred independently in the early history of metazoans and plants [Ref. (25); Figure 1]. Therefore this further supports the previously proposed idea that plant and animal NLRs are the consequence of a convergent evolution (45–47). Analysis of the phylogeny and motif combinations of the NACHT/NB-ARC domains revealed clear differences between the NACHT and the NB-ARC domains, suggesting either an ancient divergence, or an independent origin of these two domains, which happened before the divergence of eukaryotes, eubacteria, and archaebacteria (25). With the current data, both fusion events could be dated back to a period coinciding with the appearance of multicellularity (25). In this perspective, plant and animal NLRs provide an interesting example of structural and functional convergence, with a shared ability to discriminate self from non-self and to induce immune responses.

### Distinct and ancient evolutionary tracks for TNLs and nTNLs

Extending the work by Meyers et al. (48), Yue et al. (25) identified the ten most conserved motifs in NACHT and plant NB-ARC domains. This analysis revealed contrasting motif frequencies between animal NLRs and plant NLRs and further discriminates TNLs from nTNLs. This is consistent with the phylogeny based on the NB-ARC domain where plant TNLs and plant nTNLs segregate in two monophyletic clades. This result is also supported with intron phase and position analysis (18). Based on these analyses, both studies revealed a greater diversity in the nTNLs compared to the TNLs. The observed greater diversity could account for an older origin of the nTNL type compared to the TNLs, as proposed previously by Cannon et al. (49) and Meyers et al. (18). Nevertheless, the co-occurrence of TNLs and nTNLs in the bryophyte *P. patens* (25, 28), a representative of one of the most ancient land plant lineages, suggests that both NLR groups appeared in the very early history of land plants (Figure 1).

### Absence of TNLs in several plant species

Although the origins of TNLs and nTNLs seems to date back to very early land plant lineages, TNLs are known to be absent from monocots [Ref. (25, 50); Table 1]. To examine whether the other plant lineages also lack TNLs, Tarr and Alexander (31) retrieved NB-ARC sequences by using degenerate PCR combined with published datasets from diverse plant lineages, since sequences of a motif within the NB-ARC domain can be used to discriminate TNLs and nTNLs (25, 48). This study suggested the presence of TNLs in basal angiosperms and gymnosperms, whereas TNLs seem to be rare in magnoliids (Figure 1). In agreement with previous studies, no typical TNLs have been found in monocot species representing three monocot orders (31), supporting the idea that this type of NLR was lost in monocots.

TNLs are also absent from several basal eudicot families/species, such as the Lamiales, the Ranunculacea *Aquilegia coerulea* (51), and the core eudicot *Beta vulgaris* (52). Interestingly, *NRG1* (N Requirement Gene 1) genes encoding members of an atypical CNL group also appear to be absent from the plant species lacking TNLs (51). This intriguing correlation suggests a functional link between NRG1 family and TNLs (51). NRG1 was originally identified with a functional screening of immune components required for the function of N, a TNL (53). It was shown that the ADR1 (Activated Disease Resistance Gene 1) family, a very close homolog of NRG1 family, potentiates salicylic acid signaling pathway (54, 55). Since immunity mediated by many TNLs is conditioned by salicylic acid signaling (56), it is possible that NRG1 has evolved as a regulator of salicylic acid signaling especially for TNL-mediated immunity.

### Tracing back NLR function(s) in land plant evolutionary history

#### When did plant NLRs become immune regulators?

Most of the characterized plant NLRs display a classical resistance (R) gene function consisting of mediating isolate-specific effector recognition and initiating resistance responses. To date all *NLRs* classified as resistance genes belong to the angiosperms (flowering plants), summarized in Plant Resistance Gene Wiki [Ref. (57); http://prgdb.crg.eu], whereas there is no functional data available for the NLRs of other land plant taxa including gymnosperms, ferns, and bryophytes. This might be due to the lack of appropriate pathosystems that allow testing NLR functions in non-angiosperm plants. However, a few studies suggest a link between NLRs and biotic stresses in non-angiosperm plants. For example, a NB-ARC-containing gene of *P. patens* is upregulated upon abscisic acid treatment (58). In higher land plant species, this phytohormone acts in both abiotic and biotic stresses (59). It was also reported that some gymnosperm *NLRs* are differentially regulated upon interaction with microorganisms (30, 60, 61). Although these data are indicative of relatively early occurrence of NLR function in disease resistance in plant lineages, it is necessary to validate immune functions of those genes with appropriate host/pathogen systems.

#### “Atypical” NLR functions

Recent studies have revealed a role for NLRs apart from the classical R gene function. These “atypical” functions include the conditioning of broad-spectrum resistance, regulatory roles in abiotic stresses, or the role as “helper” NLR for other NLRs.

Among the *NLRs* conferring broad-spectrum resistance, Rice *Panicle blast 1* (*Pb1*) represents a well-characterized example. *Pb1* encodes a CNL (62). Pb1 confers resistance to a broad range of *Magnaporthe grisea* isolates, which contrasts with the isolate-specific resistance mediated by R genes described before. Due to its degenerate domain structure and isolate unspecific resistance phenotype, the immune mechanism mediated by Pb1 is thought to differ from the other “canonical” NLRs (62). It was recently demonstrated that Pb1 physically associates with a transcription factor, OsWRKY45, which is an essential component of the response against *M. grisea* and a prominent regulator of signaling of an important defense phytohormone, salicylic acid, in rice (63, 64). Interestingly, this physical association elevates OsWRKY45 protein amount presumably by preventing the protein degradation from an ubiquitin proteasome system (63). In addition, the successful transfer from maize to rice of Rxo1, a NLR conferring broad-spectrum resistance, suggests that the underlying resistance mechanism seems to be shared among distantly related monocotyledonous species (65).

Arabidopsis ADR1 family (ADR1, ADR1-like1, ADR1-like2) belongs to the RPW8-type of CNLs and is exceptionally conserved among various plant species including monocotyledonous and eudicotyledonous plant species (51). Because of such a high degree of conservation, much attention has been paid to this family, which might represent a conserved and potentially ancestral function. Constitutive expression of ADR1 in Arabidopsis confers drought tolerance (66, 67), indicative of its complex function beyond innate immunity.

Several NLRs are required for the functions of other NLRs. ADR1 family members are also required for PTI and ETI mediated by a distinct set of NLRs, which are dependent on salicylic acid signaling for full immune response (54). Consistent with the immune responses conferred by those NLRs, the ADR1 family is involved in a feedback amplification loop of salicylic acid signaling and its biosynthesis, cooperating with EDS1, an important immune regulator (54, 55). Another example for a helper function of NLRs is tomato NRC1. NRC1 is required for the immunity conferred by Cf-4, a non-NLR R protein. Silencing of *NRC1* in *N. benthamiana* impairs the hypersensitive response mediated by several other R proteins including two NLRs, Rx, and Mi (68). Because such “helper” NLRs are required for the functions of other NLRs, they might be involved in relaying the signal downstream of the respective innate immune sensors besides a role in defense-phytohormone pathways.

## *NLR* Gene Organization and Dynamics in the Genome

*NLR* repertoires are qualitatively and quantitatively varied among plant species (Table 1). This reflects a rapid evolution of the *NLR* family. Here we summarize insights into genomic organization and diversification of plant *NLRs*.

### *NLRs* mainly occur in clusters

*NLRs* are distributed unevenly in the genome and show a clear tendency for clustering (18, 19, 39, 41, 69). The size of clusters is rather variable, and the largest clusters contain over 10 *NLRs* in some species (19, 39). In japonica rice, the chromosome 11 alone encodes about a quarter (133 *NLRs*) of total *NLRs* (19). Overall in the rice genome, 51% of the *NLRs* reside in 44 clusters. The proportion of singletons of rice *NLRs* (24.1%) is close to that of *A. thaliana* (26.8%) (18). A similar tendency was observed in *M. truncatula* in which 49.5% of *NLRs* belong to clusters, each comprising of at least 3 *NLRs*, and 39% of *NLRs* belong to two pseudo-clusters on chromosome 3 and 6 if clustering criteria are somewhat relaxed (39). As a comparison, the human genome possesses 22–25 *NLRs* and more than 50% belong to a cluster (70). For example, 14 *NLRs* forming the *NLRP* (Nucleotide-binding oligomerization domain, leucine-rich Repeat, and Pyrin domain containing) family are present on two clusters on chromosome 11 and 19 (71). *NLRP* clusters were also found in mouse (*Mus musculus*), dog (*Canis familiaris*), and cattle (*Bos taurus*) genomes (72, 73). Therefore, clustering is a feature shared by both plant and mammalian *NLRs*.

*NLR* clusters can be divided into two types depending on the contents of NLRs: (i) homogenous clusters usually contain *NLRs* from the same type (TNL or CNL) (ii) heterogenous clusters contain a mixture of diverse *NLRs*. The former type of cluster is generated by tandem duplication, whereas the latter cluster type is derived from ectopic duplications, transpositions, and/or large-scale segmental duplications with subsequent local rearrangements (74). From an evolutionary perspective, clustering is considered as a reservoir of genetic variation (75). The size of the *NLR* clusters seems to positively correlate with the density of transposable elements on the same chromosome (34, 39). Therefore transposable elements might be involved in *NLR* evolution, possibly by increasing the genomic instability and the probability of recombination.

### *NLR* genes undergo a fast evolutionary diversification driven by combined genomic rearrangements and positive diversifying selection

The *NLR* gene family has evolved by the conjunction of duplication, unequal crossing over, ectopic recombination, or gene conversion (19, 33, 34, 39, 42, 76, 77). In addition, evidence of positive diversifying selection, an evolutionary force that favors the accumulation of mutations, is often found in *NLR*s. These processes contributed to make the *NLR* family one of the most variable gene families in the plant genomes (15, 16). Here, we further describe *NLR* evolutionary dynamics at three different scales: (i) at a genome-wide level, (ii) at a *NLR* subfamily level, and (iii) at an intragenic level.

(i) Local- and large-scale duplication events are responsible for expansion of *NLR* repertoire, but this process is partially compensated by gene contraction mechanisms (75, 78–80). As an example, *A. thaliana* has experienced two to three times whole genome duplication events, whilst NLR-encoding genes are highly underrepresented (78). These processes result in a high gene turnover, which can continuously refresh *NLR* repertoires while limiting the total number of *NLR* genes, and are together referred to as the “birth and death” process (75). Limiting *NLR* number seems to be biologically relevant, since products of *NLR* genes can come at a fitness cost (24), whereas diversity and novelty of *NLRs* can generate and maintain a broad range of resistance specificities.
(ii) The analysis of a *NLR* subfamily containing multiple *NLR* homologs revealed distinct evolutionary patterns within family members (81). This shows that evolution can shape different homologous *NLRs* in different ways. This aspect is discussed further at the section “Distinct Evolutionary Patterns in *NLR* Genes.”
(iii) Different selection mechanisms can be detected at the intragenic level, namely at regions encoding distinct NLR domains. The NB-ARC domain is generally under purifying selection, which disfavors accumulation of non-synonymous mutations, whereas positive diversifying selection is often found at region encoding the LRR domain and sometimes at the other parts of *NLR* (76, 77, 80, 82).

These mechanisms of evolution at various levels contribute to a high degree of inter- and intragenic variation of *NLR*s and account for highly species-specific *NLR* repertoires (25, 34, 76).

### Species-specific evolutionary traits and potential links to plant lifestyles

There are some species-specific features in *NLRs* evolution. For example, a higher *NLR* loss rate has been reported in maize compared to other monocot species (34), a higher degree of *NLR* clustering has been observed in *M. truncatula* (39), and a higher duplication and recombination frequency was found in two perennial woody species, wine grape and poplar (42). The latter result suggests that an increased frequency in duplication and recombination might compensate for the slower evolution rate due to a longer life cycle in some perennial species (42). In a similar manner, *NLRs* in the self-fertilizing species *A. thaliana* tend to evolve faster than in its outcrossing close relative *A. lyrata* (33, 76). Incompatible *NLR* gene interactions in offspring of crosses between particular plant individuals sometimes trigger an autoimmune-like response designated as hybrid necrosis (83). As the occurrence of hybrid necrosis is potentially greater in outcrossing species than in self-fertilizing species, hybrid necrosis might strongly influence *NLR* evolution in outcrossing species. Taken together, it is tempting to speculate that some factors like life style or reproductive fashion might influence *NLR* evolutionary processes.

### Distinct evolutionary patterns in *NLR* genes

The analysis of the *RGC2 NLR* family in diverse lettuce subspecies (*Lactuca* spp.) provided an interesting insight into the evolution of individual *NLR* genes (81). This study identified two distinct evolutionary patterns for *Lactuca NLRs*: “type I” is characterized by a “rapid innovative” mode of evolution consisting of frequent sequence exchanges with other *NLR* loci and diversifying selection, in contrast to “type II” characterized by a “conservative” mode with infrequent sequence exchange and purifying selection. This observation was also confirmed in other species, suggesting that these two mechanisms drive the evolution of a majority of plant *NLRs*. Comparison of *A. thaliana NLR* repertoire with the one of its close relatives *A. lyrata* revealed again these two types of evolutionary patterns, with the type II found in a minority of *NLRs* (<30%) present as singletons or with low copy number variation and the type I found in *NLRs* from multigenic families or clusters (33, 76). Indeed, there is a positive correlation between gene copy number and sequence exchange frequency, and similarly between cluster size and sequence exchange frequency (33, 76). This partially explains why genes in multigenic families or in clusters are more prone to diversification and why singletons are likely to remain as singletons.

Additionally, some differences might exist in the evolutionary pattern depending on the *NLR* type considered (*TNL*s or *CNLs*) although these do not show clear common trends (42, 76). For example, *TNLs* are characterized by a higher number of introns while *CNLs* are often encoded by single exons (18). Introns might give more flexibility in the recombination events. *TNLs* are therefore more prone to structure diversification via domain reshuffling.

## Structural Diversity of Plant NLRs: An Implication for Their Diversified Functions?

Beside NLRs with the conventional structure like CNL or TNL, plant genomes encode a significant number of NLRs and NLR-like proteins displaying unconventional domain composition and/or atypical domain arrangements (18, 25, 28, 39, 42). In the following paragraphs we will review the structural diversity of NLRs and NLR-like proteins in various plant species.

The “Rosetta Stone Hypothesis” proposes that when two proteins that are separate in some species are fused in another species, their fusion likely reflects a previously hidden interaction between the two seemingly non-related proteins (84). Arabidopsis RRS1 is a TNL which contains an additional WRKY domain (85). Consistent with the “Rosetta Stone Hypothesis,” a functional and physical interaction between a NLR and a WRKY transcription factor has been demonstrated in barley (86). Furthermore co-expression of individual NLR domains (i.e., N-terminal, NB-ARC, and LRR domains) can often reconstitute the full-length protein function (87–89). This suggests that the domains found in NLRs were originally separated and have then been assembled into a single multi-domain receptor during evolution. Based on the “Rosetta Stone Hypothesis,” comparison of domain structures among NLRs and NLR-relatives in various land plants and their ancestral taxa might help to detect hidden (immune) components and mechanisms constructing NLR functions.

### Tandem assembly of NLR domains

In contrast to the “typical” domain arrangements such as TNL and CNL [TIR (T), CC (C), NB-ARC (N), and LRR (L)], many “atypical” domain arrangements of plant NLRs have been reported. Some examples are TNTNL and TTNL in Arabidopsis (18), TNLT, TTNL, TNTNL, and NTNL in *M. truncatula* (39), TNLT, TNLN, TNLTN, TNLTNL, CNNL, CNLNL, and TCNL (a possible mixture of TNL and CNL) in wine grape and TNLT, TNLN, TNLTN, CNNL, TCNL in poplar (42, 90). The functional analysis of RPP2a (a TNTNL) suggests that these atypical NLRs can indeed function in disease resistance and are not just inactive chimeras (18, 91).

Tandem assemblies of the same domains are reminiscent of homotypic dimerization (oligomerization) that have been reported for several plant NLRs (89, 92–95). Apart from the TCNL arrangement with yet unidentified functions found in poplar and wine grape (42, 90), chimeras between CNLs and TNLs appear to be rare. On one hand, this might result from infrequent recombination events between *CNLs* and *TNLs* or from negative selection acting on the resulting chimeras. On the other hand, it might suggest that physical interaction between CNLs and TNLs is not functionally relevant. At least, the CNLs in monocots and some other particular plant species can function in the absence of TNLs (see Absence of TNLs in Several Plant Species). However a paradox would be the fact that some TNL functions are dependent on ADR1 family and also likely on NRG1 which both belong to the CNL type of NLRs (53, 54). Thus there might be a molecular constraint that makes fusion of two types of NLRs difficult. Alternatively, functions of TNLs might not require direct interactions with ADR1/NRG1 family.

### “Truncated” forms of NLRs

NLRs are modular proteins and therefore the reverse implication of the “Rosetta Stone Hypothesis” would suggest that separated modules or “truncated” versions of NLR could still be functional proteins. Below we discuss the phylogenetic and functional analyses, which support this hypothesis.

The genome-wide survey of Arabidopsis genes encoding either TIR- or NB-ARC-LRR-containing proteins has revealed that a significant proportion (∼28%) of those proteins are truncated forms of NLR (18). These truncated forms lack either an N-terminal domain, or the C-terminal region including the LRR with a variable part of the NB-ARC domain. *A. thaliana* genome encodes 20 TNs and 27 TXs (X indicates a domain other than CC, TIR, NB-ARC, or LRR). According to the phylogenetic analysis of the TIR-encoding genes in Arabidopsis, some large families of TNs and TXs share a common origin with TNLs, but diversified independently from the TNL family (96).

Similar truncated forms were identified in numerous other plant species including gymnosperm species, wine grape, poplar, and rice (42, 96). Phylogenetic analyses suggest that some TXs and some TNs might have orthologs in other species (42, 96, 97).

A particular family composed of atypical XTNXs was identified in Arabidopsis. BLAST searches revealed 35 homologs for these XTNXs in rice, grape, soybean, poplar, sorghum, physcomitrella, castor bean, maize, cassava, cucumis, papaya, and mimulus. These homologs have a high identity percentage. Therefore, this XTNX family seems to be highly conserved among land plants, including monocots, basal angiosperms, and magnoliids (98).

Although the function of these TN, TX, and XTNX proteins remains unclear, their diversification and conservation would suggest that at least some of these proteins do have important functions. Yet some studies on Arabidopsis TXs and TNs suggest possible roles in immunity and beyond. Arabidopsis *CHS1* encodes a TN protein which confers cold resistance by limiting chloroplast damage and cell death at low temperature. CHS1 function is achieved by regulating a PAD4-EDS1-dependent and SA-independent resistance pathway like many other TNLs (99). In several cases like CHS1, TNs appear to lack a functional NB-ARC domain (96, 99). A systematic overexpression analysis of Arabidopsis TXs and TNs in tobacco or Arabidopsis suggests that at least some TXs and TNs might function in disease resistance (98). Interestingly some TNs and TXs were shown to interact with other NLRs and/or pathogen effectors in yeast-two-hybrid assay (98).

Arabidopsis RPW8.1 and RPW8.2 (named together RPW8) possess a putative N-terminal transmembrane domain and a CC motif. This CC motif displays a high similarity with the CC found at the N-termini of a group of CNLs, sometimes referred to as RPW8-type CNLs (51, 100). RPW8 confers broad-spectrum powdery mildew resistance in Arabidopsis. RPW8 requires the phytohormone salicylic acid, EDS1, NPR1, and PAD4 for its function, suggesting that RPW8 signaling might integrate downstream components required for TNLs or basal immunity (101). RPW8 probably does not represent an ancestral function of NLRs, since RPW8 has evolved recently in Arabidopsis (102). As mentioned before, RPW8-type CNLs include ADR1 family which also displays atypical functions in and beyond innate immunity (51, 66, 67).

Truncated NLR forms can be produced by alternative splicing of full-length *NLR* transcripts. This phenomenon has already been described for diverse NLRs like L6 and N (103, 104), and those variants appear to be required for fine-tuning of the function of those NLRs (105). *RLM3* predominantly encodes a TX protein due to alternative splicing. The truncated RLM3 confers broad-spectrum resistance to necrotrophic fungal pathogens, a pathogen type that kills its host to acquire nutrients (106). Therefore, RLM3 exemplifies that, in some cases, the truncated form can be the active form.

### Variability at the central NB-ARC domain: NLRs lacking a conventional nucleotide-binding motif

Binding of ADP/ATP at the central domain (i.e., NB-ARC domain) is pivotal for plant NLR function. It has been proposed that perception of the cognate effector induces an initial conformational change of the receptor, leading to an exchange of ADP by ATP at the NB-ARC domain. The ATP binding is expected to induce subsequent conformational changes of the NLR for signal initiation (9). This model is drawn by an auto-active phenotype and loss-of-function phenotype of plant NLRs carrying non-ATP-hydrolyzing mutations and non-ATP/ADP-binding mutations at the NB-ARC domain, respectively (9, 107). However, it becomes evident that several plant NLRs confer pathogen resistance without the conventional nucleotide-binding motif (i.e., P-loop motif). For example, Rice *Pb1* encodes an unconventional NLR protein that contains two N-terminal CC domains (with a degenerate EDVID-motif) and a degenerate NB domain that completely lacks the P-loop motif (62). Interestingly, many of the NLR or NLR-like proteins which do not require a functional NB-ARC domain have non-canonical functions. For example, Pb1 confers broad-spectrum resistance to rice blast (62). The ADR1 family, as described earlier in this review, seems to have a regulatory role in biotic and abiotic stress signaling (51, 54, 55).

Altogether, these data suggest that a subset of NLRs might use an unconventional activation mechanism. Some of them also have an atypical function, suggesting that along the diversification process, some functional innovations might have arisen in these NLR families.

### Atypical domains found in the NLR structure

The study of NLRs and NLR-like proteins in various plant species has revealed that some NLRs consist of domain combinations different from the classical TNL or CNL structures. Other additional domains and other N-terminal domains have been reported. We believe that these findings might help uncovering hidden interactions and mechanisms involved in NLR function.

In the indirect recognition mode, the NLR detects effector-induced modifications of a plant protein, which is designated as “guardee,” a protein targeted by an effector, or “decoy,” a protein that mimics the target of an effector but does not have a clear biological function. It has been reported that different NLRs could monitor a guardee/decoy to detect different effector activities when effectors target the same guardee/decoy (5–8). In light of the “Rosetta stone hypothesis,” it seems plausible that a fusion event has occurred between the NLR and its cognate decoy or guardee protein. Rice RGA5 can directly bind its two cognate effectors via a non-LRR C-terminal domain. The corresponding 70 amino acids have features like a heavy metal-associated domain related to the yeast copper binding protein ATX1 (RATX1 domain) (10). Therefore RGA5 might illustrate such a fusion event between NLR and its cognate decoy or guardee. A similar RATX1 domain was found in the N-terminal domain of rice Pik-1, where it also likely contributes to effector binding (108). Therefore additional domains fused to the core NLR structure might contribute to different functions (effector recognition, NLR regulation, downstream signaling), independent of their position in the NLR backbone.

A mutation in the WRKY domain of RRS1 impairs DNA-binding and induces constitutive defense activation (109). Interestingly, the CNL MLA interacts with WRKY1/2 which also act as negative regulators of disease resistance (86). However OsWRKY45 interacting with Pb1 is a positive regulator of the Pb1-mediated immunity (63). These examples suggest diverse roles of WRKY transcription factors in plant NLR functions.

A negative regulatory role was found for the C-terminal LIM domain (named after Lin11, Isl-1, and Mec-3) of CHS3/DAR4 (110, 111). Other domains or structures have been identified at the C-terminus of some NLRs, like the Zn-metallopeptidase domain (18) or the Exo70 subunit of exocyst complex (112), but their functions remain unknown.

The N-terminal part of NLRs is typically considered as a signaling module, although it sometimes also contributes to effector recognition (1, 113), because expression of the N-terminal TIR or CC domain alone is able to trigger host cell death (51, 94, 95, 114). A variety of N-terminal domains other than TIR or CC have been identified, which are often restricted to certain taxa. CNLs in *Solanaceae* often possess an extended N-terminus. This extended N-terminus frequently contains a homologous domain, called the solanaceae domain (SD) (115). The SD domain is present in Mi-1.1, Mi-1.2, Rpi-blb2, Hero, and Prf (116). The SD domain does not resemble any known protein motif therefore its function is difficult to predict. A function of the SD domain was reported in Mi-1.2. In this case, different parts of the SD domain act as either positive or negative regulator of Mi-1.2 function (116).

More interestingly, some atypical N-termini show similarities to known structures: 6 NLRs of *P. patens* have a protein kinase (PK) domain [Ref. (28, 117); Figure 1], several NLRs of *Marchantia polymorpha* have a α/β-hydrolase domain (28), 37 NLRs of *Populus trichocarpa* have a BED-DNA-binding zinc-finger domain (42, 90). A similar zinc-finger, DNA-binding domain was found in Xa1 and in two other rice NLRs (97). The most striking example might be WRKY19/MEKK4 in *A. thaliana*, which consists of a TNL fused with a WRKY domain at its N-terminus and a MAPKKK domain at its C-terminus (WRKY-TNL-MAPKKK) (18). In addition to the known interaction between WRKYs and NLRs, these fusion events are also consistent with the reported MAPK cascade requirement for NLR function (118). Unfortunately, apart from Xa1, these atypical NLRs have not been functionally characterized (97). PK, MAPKKK, α/β-hydrolase, BED, and WRKY might represent some modules required for NLR function, either in *cis* or in *trans*. Future studies will be needed to confirm the functional link between NLR function and these modules. So far, the BED-NLRs of *P. patens* are reminiscent of the interaction of Prf with Pto kinase in tomato (115). The presence of BED and WRKY domains also suggests a possible direct role of some NLRs in transcription regulation.

## Conservation of NLR-Mediated Immunity in Plants

In addition to the aforementioned mechanisms, plant-pathogen arms race also accounts for highly species-specific *NLR* repertoires. Pathogens have evolved effectors either to increase virulence or to escape detection by the cognate NLR; in turn, plants further evolved NLRs to detect the novel effectors (119). These iterative cycles of effector and receptor adaptations drive co-evolution of many plant NLRs with pathogen effectors, thereby driving species-specific evolution of each NLR-mediated innate immune mechanisms (1). Since interfamily transfer of *NLRs* previously failed to produce stable transgenic plants with expected disease resistance, the proposed restricted taxonomic functionality of individual NLRs has been considered as a major barrier to explore *NLR* genes in unrelated plant species (120). Interfamily transfer of NLR function was shown in a few cases by co-expression of an NLR, its cognate effector and the effector target (121). However, these data are often based on transient gene expression with strong promoters and use host cell death as proxy for NLR activity. Since NLR-mediated host cell death responses can be uncoupled from NLR-mediated pathogen growth restriction in several cases (1, 122), it was unclear if plant NLRs also confer disease resistance in stable transgenic plants in phylogenetically distant species.

Recently it was shown that a subset of plant NLRs confers disease resistance across different taxonomic classes (123, 124). Our group demonstrated that a CNL designated as MLA1 (Mildew A 1) from the monocotyledonous plant barley (*Hordeum vulgare*, Poaceae) functions in the eudicot plant thale cress (*A. thaliana*: Brassicaceae) against barley powdery mildew *Blumeria graminis* f. sp. *hordei* (*Bgh*) (123). The MLA1-triggered immunity including host cell death response and disease resistance is fully retained in Arabidopsis mutant plants that are simultaneously impaired in the well-characterized defense-phytohormone pathways (ethylene, jasmonic acid, and salicylic acid). These data suggest the existence of an evolutionarily conserved and phytohormone-independent CNL-mediated immune mechanism. Similar to MLA1, co-acting Arabidopsis TNL pair, RPS4 (Resistance to *Pseudomonas Syringae 4*) and RRS1 (Resistance to *Ralstonia Solanacearum* 1) also confers resistance in cucumber (Cucurbitaceae), *N. benthamiana*, and tomato (Solanaceae) (124). Additionally the Arabidopsis *RPW8.1* and *RPW8.2* encoding truncated CNL-like proteins, confer resistance to powdery mildews in *N. tabacum* and *N. benthamiana* as in Arabidopsis (125). These results strongly imply that a subset of plant NLRs, despite their evolutionary separation, still follows a common principle in innate immunity.

Large-scale yeast-two-hybrid assays revealed that independently evolved effectors from different pathogen kingdoms (Gram-negative bacterium *Pseudomonas syringae* and obligate biotrophic oomycete *Hyaloperonospora arabidopsidis*) physically associate with the same host (Arabidopsis) proteins positioning at intersections of the host protein interaction network (126). Those proteins are designated “cellular hubs” and most of the tested hubs exhibit immune functions (126). Since the pair of RRS1-RPS4 detects three independently evolved effectors from different pathogen species (127), RRS1-RPS4 might monitor modification of a cellular hub targeted by three different effectors, enabling indirect detection. In this case, the expected cellular hub should be conserved in cucumber, *N. benthamiana*, tomato, and Arabidopsis. Indeed, such a conserved protein, EDS1, has been shown to be the target of two unrelated Pseudomonas effectors, suggesting that EDS1 might be a cellular hub guarded by RRS1-RPS4 (128, 129). Alternatively, RRS1-RPS4 might detect three cognate effectors by direct interaction as demonstrated with the co-acting rice RGA4-RGA5 (R-gene analog 4 and 5) pair, of which RGA5 physically interacts with two sequence-unrelated effectors of the rice blast fungus, *Magnaporthe oryzae* (10). At least for MLA, domain swap experiments between different MLA receptors that detect genetically diverse *Bgh* effectors, imply that recognition specificity is determined by the LRR domain (130). In addition, sequence comparison of ∼20 different MLA receptors possessing different recognition specificities revealed that diversified selection sites are predominately accumulated at the surface of the concave side of a hypothetical model of the MLA LRR structure, indicative of a direct receptor-effector interaction at the LRR domain (82, 123). Although two cognate effectors for RRS1-RPS4 have been isolated from *Pseudomonas syringae* and *Ralstonia solanacearum*, the effector of *Colletotrichum higginsianum* remains to be isolated (124, 131, 132). In addition, the cognate effector for MLA1 has not been isolated, yet. To examine how RRS1-RPS4 and MLA1 detect the cognate effectors (i.e., indirect or direct) in their native plant species and heterologous species will most likely require the identification of these effectors.

The existence of evolutionarily conserved immune mechanisms, especially downstream signaling mechanisms mediated by plant NLRs prompts a new question: how could a “conserved mechanism” have been retained during evolution despite the presumed emergence of pathogen counter arsenals that intercept this conserved signaling? It is unlikely that plant NLRs rely on a single conserved immune signaling pathway, which could be easily disarmed by pathogens. In an attempt to solve this paradox, we proposed that a single NLR could mediate immune responses via multiple signaling pathways (123), since it is difficult for pathogens to evolve an effector which simultaneously hampers multiple signaling pathways. Plants deploy NLRs at various sub-cellular locations for perception of effectors and/or initiation of immune signaling (see the review by Qi and Innes in the same issue). Thus it is tempting to speculate that entry nodes for NLR-signaling might exist at various sub-cellular locations in plants. Existence of multiple immune targets downstream of a single plant NLR (i.e., entry nodes for signaling pathways) would contribute to the robustness against rapidly evolving pathogens. This might also contribute to the conservation of plant NLR-signaling mechanism across plant species (123, 124), since a “foreign” NLR transferred with transgenic technology could have higher chances to find an entry node for downstream signaling in different plant species. Collectively, NLRs can be exploited for disease resistance breeding in a much wider range of plant species than previously thought.

## Hijacking of Plant NLR-Mediated Immunity by Pathogens

Transferring NLRs into different plant species might be a causal agent of unexpected disease, since some pathogens hijack plant NLR-mediated immunity for their proliferation. Based on nutrition modes, plant pathogens are classified into biotrophs, necrotrophs, and their intermediate, hemibiotrophs (133, 134). Biotrophic pathogens rely on living host cells for nutrition, whereas necrotrophic pathogens actively kill host cells to acquire nutrients. Hemibiotrophic pathogens are initially biotrophic and shift later to necrotrophy. Similar to biotrophic pathogens, many necrotrophic pathogens have a narrow host range infecting only one or few related plant species [summarized in Ref. (134)]. In addition to lytic enzymes and secondary metabolites, necrotrophic pathogens secrete toxins, which function as effectors to promote host cell death response. These toxins are often host-plant species-specific, thus called host-selective toxins and mediate effector-triggered susceptibility (ETS), which mirrors ETI to some extent (134).

It has been implicated that susceptibility to necrotrophic pathogens or sensitivity to their host-selective toxins is associated with *NLR* loci in diverse plant species such as Arabidopsis (135), sorghum (136), and wheat (32). These NLRs are likely maintained for resistance to other pathogens but targeted by virulent necrotrophs (137, 138). The ETS caused by the pathogenic fungus *Cochliobolus victoriae* in Arabidopsis is conditioned by a CNL, LOV1 (Locus orchestrating victorin effects 1). LOV1 is activated upon direct binding of its cognate toxin, called victorin, to a host thioredoxin related to immunity (138). Since Arabidopsis, barley, bean, Brachypodium, oats, and rice are sensitive to victorin (137, 138), the underlying principle for victorin sensitivity is expected to be conserved across plant species. However it is likely that different NLRs other than LOV1 homologs monitor the victorin action in the respective plant species, since analysis of cereal DNA databases failed to detect obvious *LOV1*-like genes (137).

Resistance to host specific necrotrophs is mediated by PTI, detoxification of toxins, loss of toxin recognitions, or restricting toxin-mediated cell death response (139). Plant NLRs seem to play minor roles in resistance to necrotrophic pathogens. However Arabidopsis *RLM3* locus, which encodes a truncated TNL lacking NB and LRR domains, confers resistance to a broad range of necrotrophs by unknown mechanisms (106).

NLR-mediated susceptibility is also observed in animal-pathogen interactions. In mouse, an NLR designated NOD2 (nucleotide-binding oligomerization domain-containing protein 2) mediates susceptibility to *Yersinia pseudotuberculosis*, a gut-living bacterial pathogen that disrupts the interstitial barrier to invade host cells (140). Similar to plant pathogens, *Y. pseudotuberculosis* delivers a set of effectors through the type III secretion system for virulence. Among the effectors, YopJ, an acetyl-transferase, mediates the intestinal barrier dysfunction by redirecting NOD2 signaling. YopJ acetylates RICK (Rip-like interacting caspase-like apoptosis-regulatory PK), an immediate downstream target of NOD2, resulting in reduced binding affinity of RICK to NOD2. As a consequence, NOD2 is able to form a complex with caspase-1 other than RICK, resulting in higher IL-1β production. This appears to increase the intestinal permeability for the bacterial invasion (140). Consistently, Crohn’s disease-associated NOD2 mutations found in ∼20% of healthy white individuals are likely maintained to protect the host from systemic infection by common enteric bacteria (141). Similar to *Y. pseudotuberculosis*, *Salmonella enterica* subspecies trigger host immune responses (i.e., inflammation) to obtain a niche in the already established gut microbial community (142), suggesting that induction of inflammatory responses might be a common strategy for pathogenesis of enteric bacteria.

Thus host immune response is sometimes beneficial for pathogens in plants and animals. Plant pathogens might also exploit host immune mechanisms to compete with host associating microorganisms. Plants and animals deploy an array of NLRs to fight against pathogens, whilst deployment of NLRs must be tightly balanced. Otherwise, these could be exploited by pathogens. Such a constraint might also contribute to shaping the current repertoires of NLRs in plants and animals.

## Plant NLRs Regulating Transcription

Apart from the host cell death response, NLR action is often associated with transcriptional changes. Here we review the emerging picture how NLRs actively participate in transcriptional regulation in plants.

It has been shown that transcriptional differences in resistant vs. susceptible interactions are rather quantitative than qualitative in several cases. This implies that NLRs amplify or sustain defense-related gene expression mediated by pattern-recognition receptors (123, 143–147). Transcriptome analysis comparing gene expression mediated by a TNL and a CNL, each recognizing different effectors from the same pathogen, identified a common set of target genes. This indicates that the underlying mechanism for transcriptional regulation might be shared by both types of NLRs (148). Recent studies start to unravel how NLR action is converted to transcriptional reprograming.

Recognition of the cognate effectors by plasma membrane-associated CNLs RPS2 (Resistance to *Pseudomonas Syringae* 2) and RPM1 (Resistance to *Pseudomonas Syringae* pv *Maculicola* 1) results in transcriptional reprograming (144, 149), indicating a mechanism that relays signals from the plasma membrane to the nucleus. To uncouple ETI from PTI with a synchronized homogeneous cell population, Gao et al. (150) used an Arabidopsis protoplast system, in which the cognate effectors for RPS2 or RPM1 are expressed under an inducible promoter. Genome-wide transcriptome analysis with the protoplast system identified *WRKY46* as an early marker gene shared in RPS2- and RPM1-mediated signaling. Since chemical inhibitors affecting various Ca^2+^ channels suppressed the effector-mediated *WRKY46* promoter activation, potential involvement of Ca^2+^-dependent protein kinases (CPKs) were examined. A genetic and biochemical screen identified a group of Ca^2+^-dependent PKs (CPK 4, 5, 6, and 11), acting as signaling mediators between the NLRs and the transcription factors WRKY8, WRKY28, and WRKY48. Those WRKYs are proposed to regulate gene expression downstream of RPS2 and RPM1. Notably, another group of CPKs (CPK1 and 2) appears to be involved in host cell death response rather than transcriptional reprograming, suggesting the existence of a bifurcated CPK-dependent signaling pathway mediating distinctive NLR-triggered immunity outputs (i.e., cell death and transcriptional reprograming). However, it still remains unclear how RPS2 and RPM1 activate the set of CPKs. So far, a direct interaction between the CPKs and RPS2 or RPM1 was not detected (150). Potential players in the RPS2 or RPM1-CPK signaling cascade might be CNGCs (cyclic nucleotide-gated ion channels), a family of putative Ca^2+^ channels, some of which are involved in plant immunity (151, 152). However, the mechanistic link between NLRs and CNGCs remains unknown.

Signaling relay via a mediator such as CPK might be one mechanism by which membrane-associated NLRs regulate transcriptional reprograming. However, recent work indicates that some soluble NLRs participate in an even shorter signaling pathway. Localization into the nucleus has been shown for several NLRs. When excluded from the nucleus by fusion with a nuclear exclusion signal, immunity mediated by the nucleo-cytoplasmic barley MLA10 (CNL) is compromised (86). Similarly, nuclear exclusion of the nucleo-cytoplasmic N (TNL) resulted in compromised immunity in *N. benthamiana* (153). Disruption of the nuclear localization sequence of Arabidopsis RPS4 (TNL) resulted in impaired immunity toward *Pst* DC3000 expressing its cognate effector (154). Together, these data point toward a nuclear function of a subset of NLRs.

Recent studies have started to elucidate the activity of nuclear-localizing NLRs. Following up on the demonstration that barley MLA10 interacts with HvWRKY1 and HvWRKY2, negative regulators of immunity, Chang et al. (155) elucidated the mechanism by which this interaction results in immunity. They demonstrated that the CC domain of barley MLA10 interacts not only with the aforementioned repressors but also with the transcriptional factor HvMYB6, a positive regulator of immunity. Strikingly, only the active form of MLA10 is able to bind HvMYB6, which is sequestered by HvWRKY1 in the absence of the activated MLA10. The interaction through the MLA CC domain prevents WRKY1 from interacting with HvMYB6, thereby allowing HvMYB6 binding to the corresponding *cis*-element. The MLA10-HvMYB6 complex, in turn, greatly enhances transcription downstream of the *cis*-element compared to HvMYB6 alone in a transient assay. While this interaction greatly adds to our understanding of MLA function in barley, it cannot explain the conserved function of MLA1 in Arabidopsis (123), since HvMYB6 is a highly monocot-specific transcription factor (155).

Pb1, a rice CNL, has also recently been shown to interact with the transcription factor OsWRKY45, likely leading to transcriptional reprograming. However, in contrast to the MLA-HvMYB6 interaction, the transcriptional activity is regulated via OsWRKY45 abundance, since Pb1 protects OsWRKY45 from degradation upon pathogen attack (63).

A third example aiding in our understanding of NLR nuclear activity is the interaction of N with the transcription factor SPL6 (SQUAMOSA PROMOTER BINDING PROTEIN-LIKE 6) in *N. benthamiana* (156). The association of N and SPL6 at subnuclear bodies occurs only in the presence of the cognate effector. A genetic requirement for SPL6 was shown in *N. benthamiana* for N-mediated disease resistance as well as in *A. thaliana* for RPS4-mediated immunity. A number of RPS4-mediated defense responsive genes are differentially regulated upon *AtSPL6* silencing (156).

Close re-examination of yeast-two-hybrid data generated by Mukhtar et al. (126) provides further support of NLR-transcription factor interaction as a more common mechanism of NLR actions. Mukhtar et al. (126) tested interactions using as bait N-terminal domains of Arabidopsis CNLs and TNLs, which have previously been demonstrated to function as minimal signaling domains in some cases (94, 95), and as prey full-length constructs of ∼8,000 immune-related genes including transcriptional regulators. Strikingly, of those NLRs showing interactions, the majority interacted with one or more transcriptional regulators. Furthermore, these interactions could be found for both CNLs and TNLs. Interaction between transcriptional regulators and NLRs has already been demonstrated too, for example the interaction of the transcriptional co-repressor TPR1 (Topless-related 1) with the Arabidopsis TNL SNC1 (157).

Taken together, these studies draw an emerging picture in which nuclear localized NLRs mediate transcriptional reprograming via interaction with transcription factors in various plants species. Interaction with transcriptional regulators appears not to be limited to one subclass only or to just a few specialized NLRs. Instead, this type of interactions might be a more common phenomenon, implying a possible general mechanism of direct regulation of transcriptional reprograming via plant NLRs. Transcriptional regulation via NLRs also occurs in animals. Two well documented NLRs, CIITA and NLRC5, both regulate a set of genes, MHC class I and class II genes, by recognizing specific *cis*-elements and recruiting a group of transcriptional regulators (158, 159). The protein complex formed is known as enhanceosome (160, 161). It remains to be proven whether NLRs in plants also form such large order complexes or modulate transcription by interacting with only a few transcriptional regulators at a time.

## Structural Insight into Auto-Inhibition Mechanism of NLRs

Very recently the first crystal structure of an NLR monomer (mouse NLRC4) in its inactive state was resolved (162). The structure revealed the presence of multiple “security locks,” coordinated by several and distinctive intra-domain interactions to keep the receptor in an inactive state. These locks prevent the receptor from homo-oligomerization driven by associations through the central domain. The observed intra-domain interactions cluster in close proximity of the potential ligand-binding pocket, which is primarily shaped by the LRR domain together with the other domains (162). Thus, it is proposed that ligand-binding at the pocket could release the multiple locks all at once, enabling a subsequent conformational change of the receptor (e.g., ADP-ATP exchange, oligomerization). Interestingly, the structure and the experimental evidence suggest that ADP-binding at the P-loop motif also contributes to auto-inhibition of the receptor. However, the inhibition mechanism seems to be distinctive from that mediated by the other intra-domain interactions, since the position of ADP in the crystal is distant from the pocket (162). Unlike animal NLRs, plant NLRs lack the HD2 sub-domain (also known as ARC3 sub-domain) in the central NB-ARC domain (14), and general applicability of the central domain mediated homo-oligomerization of plant NLRs upon receptor activation is unclear.

The LRR domain of plant NLRs is also involved in forming “security locks” by cooperating with the other domains in the absence of pathogens (93, 163–166). A structure-function analysis combined with docking simulations of structural models of the NB-ARC and the LRR domains identified regions that determine intra-domain interactions in two CNLs, Rx1 and Gpa2 (166). At least in the case of these two highly homologous CNLs, the association between the N-terminal repeats of the LRR domain and a small region of the ARC2 domain are sufficient to keep these NLRs in an inactive state, whilst the rest of C-terminal repeats of the LRR domain act as the major determinant of the effector recognitions (166). Thus it is proposed that detection of the cognate effectors at the C-terminal repeats of the LRR domain disrupts the intra-domain interaction to activate the receptor (166).

## Conclusion and Perspective

Over the past few decades, many NLRs and NLR-like proteins were isolated from plants and animals and their functions have been extensively studied. The development of new technologies has further accelerated research on NLR biology. For example, deep sequencing technology offers more opportunities to conduct comparative genome-wide analyses of *NLRs* in various species. Whole-transcriptome analysis at single transcript level combined with ChIP-seq analysis (chromatin immunoprecipitation followed by sequencing) allows to uncover underlying mechanisms for NLR functions in the nucleus. Furthermore, structural biology provides in-depth understanding of mechanistic insights into NLR actions. Nevertheless, a balanced combination of those technologies and “classical” genetics and biochemical studies are important to unravel the principle of NLR functions.

As we discussed above, a plant NLR might initiate downstream signaling by connecting to multiple signaling targets rather than through a single evolutionarily conserved target. Despite a lack of direct experimental evidence to date, putative compartment-specific activities of plant NLRs, particularly in the cytoplasm and nucleus (129, 167), suggest that a single NLR interacts with structurally different downstream components to initiate immune responses in different compartments. Thus, it might be possible that a second, third, or even more downstream signaling layers exist for a given NLR, including several interacting components that might constitute “as a whole” the downstream innate immune mechanism. Finally, we imagine that comprehensive knowledge of NLR actions would allow the design of synthetic NLRs in order to control pathogens and manipulate NLR functions even beyond innate immunity.

## Conflict of Interest Statement

The authors declare that the research was conducted in the absence of any commercial or financial relationships that could be construed as a potential conflict of interest.
